# Gene expression of pea aphid (Hemiptera: Aphididae) salivary effectors changes based on feeding duration and plant species

**DOI:** 10.1093/jisesa/ieag056

**Published:** 2026-06-19

**Authors:** Lani Irvin, Jinlong Han, Vamsi Nalam, Cecilia Tamborindeguy, Susana Karen Gomez

**Affiliations:** Department of Biological Sciences, University of Northern Colorado, Greeley, CO, USA; Department of Agricultural Biology, Colorado State University, Fort Collins, CO, USA; Department of Agricultural Biology, Colorado State University, Fort Collins, CO, USA; Department of Entomology, Texas A&M University, College Station, TX, USA; Department of Biological Sciences, University of Northern Colorado, Greeley, CO, USA

**Keywords:** aphids, effectors, transcripts, plants, feeding

## Abstract

Aphids are agricultural pests that damage crops directly through feeding and indirectly by transmitting plant viruses. Central to their success are salivary effectors, secreted proteins that suppress plant defenses and facilitate nutrient uptake. Nonetheless, numerous salivary effectors remain uncharacterized to date. It is unclear whether effector gene expression patterns vary in response to factors such as feeding duration, aphid age, tissue type, and host plant. To address current knowledge gaps, we reared pea aphids, *Acyrthosiphon pisum* (Harris) (Hemiptera: Aphididae), and 2 host plants, barrel medic (*Medicago truncatula* Gaertn.) and broad bean (*Vicia faba* Linnaeus), under a controlled environment. Next, we quantified the gene expression patterns of 2 well-characterized (*C002* and *Armet*) and 2 less studied aphid salivary effectors (*Apolipophorin* and *ACYPI006346*). The results show that salivary effector gene expression declines by 72 h when switching hosts (*V. faba* to *M. truncatula*), although some effectors display fluctuating patterns between specific time points (24 vs 48 h and 48 vs 72 h) when switching hosts (*V. faba* to *M. truncatula*) or not (*M. truncatula* to *M. truncatula*). One-day-old aphids consistently exhibit higher gene expression levels than older individuals when fed on a single host (*V. faba* to *V. faba*). These findings highlight the importance of considering temporal dynamics, developmental stage, and host context in studies of aphid effector biology. Our findings lay a methodological foundation that supports the design of more consistent and informative transcriptomic experiments on aphid–plant interactions.

## Introduction

Phloem-feeding hemipterans such as aphids use piercing–sucking mouthparts to reach the phloem sieve elements without causing major cellular damage. During the feeding process, aphids secrete gelling and watery saliva, each serving distinct roles. The gelling saliva primarily protects the stylet from plant apoplastic defenses. Intracellular secretion and re-ingestion of watery saliva during probing allow aphids to assess when the phloem sieve tube element is reached and to evaluate the host’s nutritional quality and defensive compounds. Once the phloem sieve tube element is punctured, watery saliva is secreted to manipulate plant defenses and facilitate sustained feeding (Giordanengo et al. 2010, Twayana et al. 2022). In addition to these salivary secretions, aphids deploy a suite of salivary secreted proteins, known as effectors, which are crucial for manipulating plant cellular processes. In the case of pea aphids, 3,603 candidate effector genes have been reported in salivary glands (Boulain et al. 2018). The secretion of effectors through the stylets into plant tissues modulates plant physiology to the insect’s advantage by suppressing immune responses and facilitating phloem sap consumption, allowing aphids to feed and propagate with minimal detection while evading plant defenses, which is critical for their survival and reproduction (Miles 1999, Cherqui and Tjallingii 2000, Powell 2005, Tjallingii 2006, Bos et al. 2010, Hogenhout and Bos 2011, Will et al. 2012, Dommel et al. 2020). Given the intricate relationship between aphids and their host plants, different suites of effector genes are likely expressed at different moments during feeding or in association with different host plants.

Despite significant advances in omics technologies that have greatly facilitated the identification and characterization of candidate aphid salivary effectors, it often is assumed that the gene expression patterns of these effectors vary in response to different host plants, feeding durations, aphid tissue types (salivary gland, whole heads, whole bodies, alimentary tract, brain, etc.), and aphid developmental stages (Carolan et al. 2009, 2011, Bos et al. 2010, Lu et al. 2016, Thorpe et al. 2016, 2018, Boulain et al. 2018, 2019, Dommel et al. 2020). Recent studies have highlighted that the gene expression and activity of aphid salivary effectors can exhibit diurnal rhythmicity and may be modulated by environmental and physiological factors, further complicating our understanding of their roles in aphid–plant interactions (Han et al. 2024).

Feeding behavior and salivary composition in insects are subject to numerous biotic and abiotic factors, requiring careful experimental design. For example, both salivary gene expression and effector activity display diurnal variation, making it essential to collect samples at consistent times to avoid confounding effects related to circadian rhythms rather than experimental treatments. Dissecting salivary glands, while offering tissue specificity, can delay sample collection and potentially compromise RNA integrity. As an alternative, researchers may assay whole heads or bodies, but this approach may reduce sensitivity for detecting salivary gland-specific gene expression due to tissue heterogeneity. Additionally, the inclination of insects to feed can vary; thus, it is important to standardize or record the feeding duration for experiments. Short starvation periods are sometimes used to stimulate feeding, but extended starvation may cause dehydration, alter feeding behavior, and artificially induce the expression of stress-responsive genes (Ramírez and Niemeyer 2000). Together, these factors underscore the importance of tightly controlled sampling protocols when assessing salivary effector gene expression, as minor deviations in handling, timing, or feeding conditions can introduce biological noise and obscure treatment-specific responses.

This study aims to elucidate the gene expression patterns of 4 aphid salivary effectors in response to 2 distinct host plants and varying feeding durations. To address this, we conducted a series of experiments, each designed with different independent variables such as host plant species and feeding duration. We selected *Medicago truncatula* and *Vicia faba* as host plants because both are well-established systems for aphid–plant interaction studies (Klingler et al. 2005, Elzinga and Jander 2013, Guo et al. 2014, Kamphuis et al. 2016, Lu et al. 2016, Stewart et al. 2016, The International Aphid Genomics Consortium 2018). *Medicago truncatula* is a model legume with extensive genomic resources and well-characterized defense responses, while *V. faba* serves as a universal host for all pea aphid biotypes and is agriculturally significant. Their use allows for robust comparative studies of aphid gene expression and plant defense mechanisms. Importantly, the choice of *M. truncatula* and *V. faba* is supported by previous research that used these 2 legumes to investigate pea aphid effector–plant interactions, justifying their selection over other legumes (Pan et al. 2015, Lu et al. 2016, Boulain et al. 2018, 2019, Thorpe et al. 2024). These experiments were conducted to systematically establish optimal experimental conditions for subsequent transcriptome analyses (unpublished data). The primary goal was to establish whether the gene expression of aphid salivary effectors fluctuates or remains stable across varied experimental conditions.

In this study, we investigated the temporal gene expression profiles of 4 aphid salivary effectors: *Armet*, *C002*, *ACYPI006346*, and *Apolipophorin*. The selection of 2 well-characterized effectors, such as *Armet* and *C002*, was based on previous functional studies demonstrating their critical roles in aphid feeding and survival, with *Armet* (also known as MANF) being involved in modulating plant calcium signaling and suppressing plant defense responses, and *C002*, which is expressed in the primary salivary gland, being essential for sustained phloem feeding (Mutti et al. 2006, 2008, Wang et al. 2015, Cui et al. 2019, Fu et al. 2023, Xue et al. 2024). *ACYPI006346* was selected as a marker for salivary glands to verify successful salivary gland dissections and to assess potential dilution effects of salivary transcripts when head tissues were used instead. *ACYPI006346* is upregulated in the salivary glands of *Acyrthosiphon pisum*, particularly in cell types 5 and 7 of the principal salivary glands (Pan et al. 2015). To our knowledge, *Apolipophorin* has not been studied in aphids (Nicolis et al. 2022). This gap prompted us to search for the *Apolipophorin* ortholog in the pea aphid, given its roles in innate immunity, lipid transport, and other immune-related functions in chewing insects (Zdybicka-Barabas and Cytrynska 2011, 2013, Pan et al. 2015). Although several aphid salivary effectors have been functionally characterized, each playing distinct roles in facilitating feeding and modulating plant defenses, comparative studies examining the expression dynamics of these genes under different biological and experimental conditions remain limited.

Here, we systematically investigate how salivary effector gene expression in *A. pisum* is shaped by feeding duration, host plant, and aphid developmental stage. Using *M. truncatula* and *V. faba* as hosts, we quantified the gene expression of 4 salivary effectors (*Armet*, *C002*, *ACYPI006346*, and *Apolipophorin*) across 4 independent experiments. To interpret the regulatory mechanisms underlying variation in salivary effector gene expression, we proposed 3 hypotheses informed by established physiological, molecular, and behavioral adaptations in aphids (Klingler et al. 2005, Kamphuis et al. 2016, Stewart et al. 2016). First, because host plant transitions impose immediate chemical and defensive challenges, shifting pea aphids from *V. faba* to *M. truncatula* is expected to trigger a rapid, transient upregulation of key salivary effector genes (*Armet*, *C002*, *ACYP1006346*) during early host acceptance, followed by a partial decline toward a new steady state as aphids establish sustained feeding on the new host. Second, because plants deploy localized, time-dependent inducible defenses, we expect that prolonged feeding on *M. truncatula* will drive dynamic changes in aphid salivary effector gene expression, reflecting progressive physiological adaptation to the new host. Third, because rapid early-stage growth and colonization may require strong suppression of initial host barriers, we hypothesize that younger aphids will exhibit higher salivary effector gene expression than older aphids, reflecting distinct metabolic and developmental demands early in life. Our overarching goal was to identify the conditions that modulate salivary effector gene expression to guide transcriptome experimental design. These insights will support researchers studying salivary effector ­biology, aphid physiology, and host specialization across hemipteran insects.

## Materials and Methods

### Plant Growth Conditions

*Medicago truncatula* (Jemalong A17) seeds were sterilized and germinated following established protocols (Maurya et al. 2018). Ten to 12 *M. truncatula* seedlings per pot were initially transplanted into azalea pots (10.2 × 6.9 cm; diameter × height) with a 1:1:1 mix of concrete sand, gray breeze (crusher fines), and squeegee (rounded gravel mix) (Pioneer Sand Company, Greeley, Colorado), and grown in a PGC Flex plant growth chamber (Conviron, Winnipeg, Canada) under a 16 h ­photoperiod at 24 °C and 40% relative humidity, with light from fluorescent and halogen bulbs (285 to 290 µmol m^−2^ s^−1^). Soil substrates were autoclaved for 60 min, twice, at 21 psi. *Medicago truncatula* plants with 2 to 3 trifoliolate leaves were transplanted into square pots (8.9 × 7.6 cm; diameter × height) to serve as experimental plants. They were watered daily with 50 ml of Milli-Q water and fertilized biweekly with 50 ml of one-half strength modified Hoagland’s Solution (100 μM P) (Amon and Hoagland 1940).

Broad bean (*V. faba*) (Broad Windsor fava bean; Amazon) seeds were soaked in deionized water for 24 h prior to planting. These seeds were then sown in azalea pots filled with sterilized Lambert LM6 soil (American Clay Works, Denver, Colorado) and maintained at room temperature (∼24 °C) under a 16 h photoperiod. *Vicia faba* plants were fertilized weekly with 50 ml of Miracle-Gro All Purpose Plant Food (0.8 g/liter).

### Aphid Rearing and Sample Collection

Pea aphids (*A. pisum* LSR1) were reared on the universal host *V. faba* under laboratory conditions (16 h light at 27 °C, 8 h dark at 24 °C). To synchronize cohorts, adult aphids were confined on plants for 24 h to produce nymphs, which were then aged for 6 days (approximately fourth-instar nymphs) before use (Stewart et al. 2016, Olson et al. 2022). All sample collections occurred at 2 PM (8 h after the light is on) to minimize the influence of circadian rhythms on the experimental results (Goodspeed et al. 2012). To guarantee that all biological replicates were harvested precisely at 2 PM, aphid infestation start times were strictly back-calculated and staggered based on the designated feeding durations. For instance, in experiment B, aphids assigned to the 6 h feeding duration were introduced to the experimental plants at 8 AM on the day of harvest, while those assigned to the 24 and 48 h durations were introduced at 2 PM 1 and 2 days prior, respectively. Additionally, each treatment replicate was harvested simultaneously to ensure consistency across temporal variables. After the specified feeding duration, aphids were collected and immediately killed with 70% ethanol; the ethanol was removed, and aphids were rinsed with sterile Milli-Q water. In experiments A and B, the ethanol was removed, aphids were rinsed with sterile Milli-Q water, preserved in 1.5 ml RNAlater (Thermo Fisher Scientific, Waltham, Massachusetts, United States), and stored at 4 °C per manufacturer’s protocol until RNA extraction was performed. In experiments C and D, aphid heads were dissected, frozen, and ground in liquid nitrogen and stored at −80 °C until RNA extraction was performed.

### Experimental Setup

**Fig. 1.**
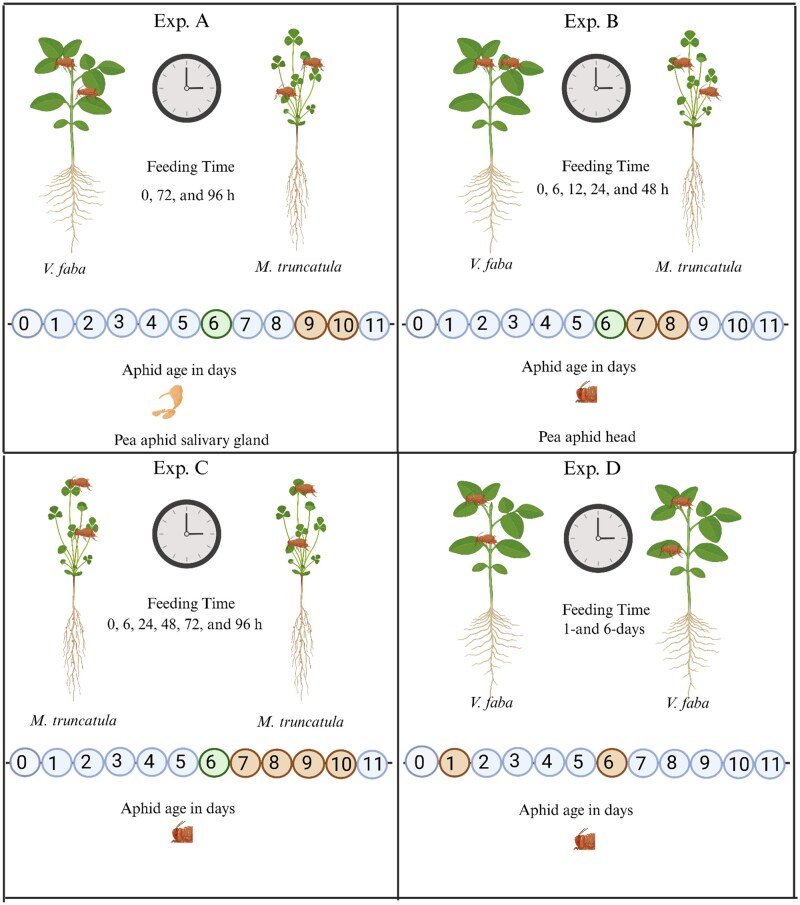
Schematic representation to investigate gene expression of aphid salivary effectors. Schematic summary of the 4 independent experiments assessing salivary effector gene expression based on aphid tissue type, host plant, aphid age, and feeding duration. Experiments included: A) salivary glands from pea aphids (*Acyrthosiphon pisum*) reared on *Vicia faba* and switched to *Medicago truncatula* sampled at 0, 72, and 96 h; B) heads from pea aphids reared on *V. faba* and switched to *M. truncatula* sampled across a time course (0 to 48 h); C) heads from aphids maintained continuously on *M. truncatula* (0 to 96 h); and D) heads from 1- and 6-day-old pea aphids maintained continuously on *V. faba*. For the 0 h time point, pea aphids were taken directly from the rearing plant (*V. faba* or *M. truncatula*). Each experiment assessed gene expression of 3 aphid salivary effector genes: *Armet*, *C002*, and *ACYPI006346*. Gene expression of *Apolipophorin* was assessed in experiments C and D. Created in BioRender) https://BioRender.com/y90m431

To investigate how feeding duration, host plant, aphid age, and tissue type influence the expression of aphid salivary effector genes, we conducted 4 independent experiments (A to D) using synchronized *A. pisum* cohorts. For all experiments, biological replicates consisted of pooled aphid tissues, with samples collected at 2 PM to minimize circadian effects. Each treatment included 3 biological replicates, and all aphids were 6 days old at the start of infestation unless otherwise specified. A schematic overview is provided in Fig. 1, and the experimental parameters are summarized in Table 1. *Medicago truncatula* used in the experiments were of similar age, ranging from 46 to 60 days at the time of harvest, and the aphids were synchronized to ensure that their age was consistent across all experiments. This methodological rigor minimizes variability and strengthens the reliability of our findings.

**Table 1.** Summary of experimental conditions

Experiment	Host plant(s) (rearing → experimental)	Feeding duration	Aphid age at collection (days)	Tissue type	Starvation prior to feeding	Sample size per replicate
**A**	*Vicia faba* → *Medicago truncatula*	0, 72, 96 h	6, 9, and 10	Salivary glands	No	20 salivary gland pairs
**B**	*Vicia faba* → *Medicago truncatula*	0, 6, 12, 24, 48 h	6, 7, and 8	Whole heads	Yes (1 h)	20 heads
**C**	*Medicago truncatula* → *Medicago truncatula*	0, 6, 24, 48, 72, 96 h	6, 7, 8, 9, and 10	Whole heads	Yes (1 h)	5 heads
**D**	*Vicia faba* → *Vicia faba*	1, 6 days	1 and 6	Whole heads	No	20 heads

All experiments used synchronized aphids collected at 2 PM to control circadian effects. *Medicago truncatula* plants used in experiments A to C ranged from 46 to 60 days at the time of harvest. Three biological replicates were included per treatment. Aphid feeding was conducted under standardized growth chamber conditions (16 h light/8 h dark, 24 °C).

Experiment A assessed whether reinstallation on a novel host plant alters effector gene expression in salivary glands. Aphids were reared on *V. faba* and then transferred to *M. truncatula* for feeding durations of 0 (taken directly from *V. faba*), 72, or 96 h. Salivary glands were dissected from aphids at each time point, with 20 gland pairs pooled per replicate (Atamian et al. 2013). Aphids were stored in 70% ethanol until their respective pools were dissected. Before dissection, they were rinsed with 1× phosphate‑buffered saline (pH 7.4) using fine tweezers and kept on ice until the salivary glands were removed. Dissected salivary glands were immediately frozen in liquid nitrogen. Approximately 1 h was required to dissect 20 salivary glands. This tissue was chosen because it represents the most direct site of effector gene transcription, particularly for low-abundance transcripts.

Experiment B focused on early-stage transcriptional changes following host plant switching. Aphids reared on *V. faba* were starved for 1 h to stimulate synchronized feeding and then transferred to *M. truncatula* for 0 (taken directly from *V. faba* and starved), 6, 12, 24, or 48 h of feeding. Whole heads were collected (20 pooled heads per replicate), enabling moderate-throughput sampling while retaining salivary gland tissue (Atamian et al. 2013). This approach allowed us to capture short- and intermediate-term expression dynamics without requiring salivary gland dissection.

Experiment C examined effector gene expression during continuous feeding on a single host plant. Aphids reared on *M. truncatula* were starved for 1 h to stimulate synchronized feeding and then allowed to feed for 0 (taken directly from *M. truncatula* and starved), 6, 24, 48, 72, or 96 h on new *M. truncatula* plants. Heads were dissected and pooled (5 heads per replicate) (Bos et al. 2010). This design enabled analysis of time-dependent gene expression without the confounding effects of host plant switching.

Experiment D evaluated the effect of aphid age on salivary effector expression. Adult aphids reared on *V. faba* were allowed to reproduce on new *V. faba* plants, and nymphs were sampled either 1 day or 6 days after birth. The adults were removed after 1 day. Heads from 20 individuals were pooled per replicate (Atamian et al. 2013). This experiment tested whether younger aphids exhibit higher effector gene expression, reflecting their elevated metabolic demands and earlier developmental stage.

### RNA Isolation and cDNA Synthesis

Total RNA extraction from aphid tissue (salivary gland or whole head) was performed using the PureLink RNA Mini Kit (Thermo Fisher Scientific), following the manufacturer’s instructions. The extracted RNA was subjected to DNase treatment using Turbo DNase (Thermo Fisher Scientific) and incubated for 40 min at 37 °C. After DNase treatment, the samples underwent a purification process according to a modified protocol (Maurya et al. 2018, Olson et al. 2022). RNA quantification was conducted using the Thermo Scientific NanoDrop 2000C spectrophotometer. The integrity of RNA and the exclusion of genomic DNA were verified through agarose gel electrophoresis in 1% hydrogen peroxide (Pandey and Saluja 2017). For cDNA synthesis, 1 μg of total RNA was reverse transcribed using SuperScript IV (Thermo Fisher) per the manufacturer’s protocol. The quality of the synthesized cDNA was assessed by semi-quantitative PCR targeting the Succinate Dehydrogenase B (*SDHB*) gene (Yang et al. 2014), and the amplicons were visualized on a 2% (w/v) agarose gel in 0.5× TAE buffer.

### RT-qPCR and Reference Gene Selection

RT-qPCR was used to quantify relative expression levels of 4 aphid salivary effectors: *Armet*, *C002*, *Apolipophorin*, and *ACYPI006346*. *Apolipophorin* was not measured in experiments A and B due to RNA limitations. Primers for both target and reference genes were either selected from the literature or designed using NCBI Primer-BLAST. Primer sequences, gene IDs, efficiencies, and annealing temperatures are provided in Supplementary Table S1. Each 10 μl qPCR reaction included 1 μl of 1:5 diluted cDNA, 5 μl of Power SYBR Green Master Mix (Thermo Fisher Scientific), 1 μl of forward/reverse primer mix (3 μM), and 2 μl of nuclease-free water. Reactions were run on a Bio-Rad C1000 Touch Thermal Cycler with the following conditions: initial denaturation at 95 °C for 10 min, followed by 40 cycles of 95 °C for 15 s, and annealing/extension at the appropriate temperature (55 to 60 °C) for 1 min. Melt curve analysis was performed from 65 to 95 °C in 0.5 °C increments to confirm amplification specificity. For each biological replicate, the 2 technical replicates with comparable Cq values were averaged to produce a single Cq value. Of the 7 reference genes tested, 4 reference genes (*SDHB*, *Rsp20*, *Actin*, and *TpL27*) were determined to be stable under our experimental conditions using the NormFinder Software (Yang et al. 2014). For each experiment, we selected the most stable reference gene based on its Cq values and primer efficiency (Supplementary Table S1). Relative gene expression was calculated using the Pfaffl method (Pfaffl 2001) to account for differences in amplification efficiencies between target and reference genes.

### Statistical Analysis

Statistical analyses were conducted utilizing either RStudio (R Core Team 2023) or the data analysis tools available in Microsoft Excel. Data from RT-qPCR experiments were Log_10_-transformed to meet the assumptions of normality and homogeneity of variances required for parametric testing (Taylor et al. 2019). The transformed data were assessed for normality and homogeneity of variances using the Shapiro–Wilk test and Levene’s test in RStudio. The analysis of relative gene expression was performed using one-way Analysis of Variance, complemented by post-hoc Tukey’s honestly significant difference (HSD) test or Welch 2-Sample *t*-test as appropriate. All *P* values <0.05 were considered statistically significant. The outcomes for each treatment were graphically displayed using RStudio (R Core Team 2023). The raw data files, R-scripts for data analysis and graphing, and data output are stored on the repository Dryad (https://doi.org/10.5061/dryad.kwh70rzkg).

## Results

### Gene Expression of Pea Aphid Salivary Effectors Decreases at Specific Feeding Times When Aphids Switch Hosts

**Fig. 2.**
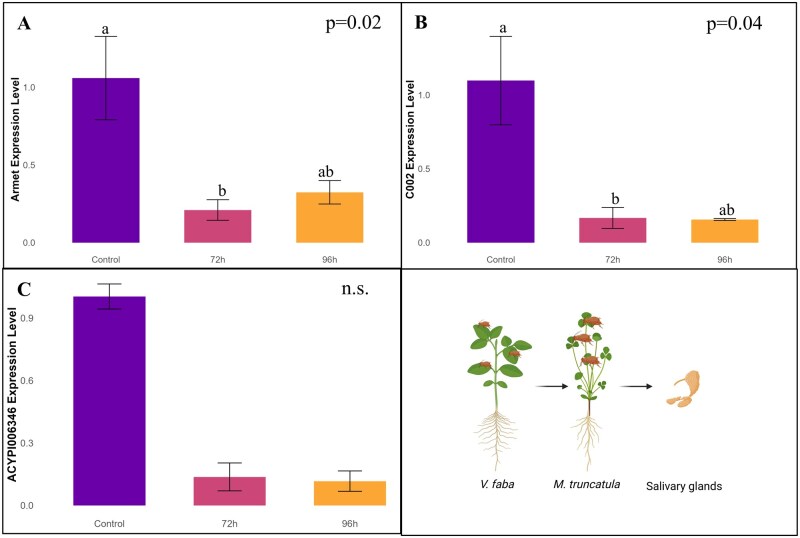
Aphid salivary effector gene expression in dissected salivary glands after host switch. Relative gene expression of A) *Armet*, B) *C002*, and C) *ACYPI006346* in salivary glands of pea aphids (*Acyrthosiphon pisum*) reared on *Vicia faba* and switched to *Medicago truncatula* collected at 0 (taken directly from *V. faba*), 72, and 96 h. Relative expression was normalized to the reference gene *SDHB*. Data represent mean ± SEM (3 biological replicates). Each biological replicate consisted of 20 dissected salivary gland pairs. Letters denote significant differences across time points (Tukey’s HSD, *P *< 0.05).

In experiment A, aphids reared on *V. faba* were transferred to *M. truncatula* for 0 (taken from *V. faba*), 72, or 96 h. Gene expression of both *Armet* (*F*_(2,6)_ = 8.05, *P* = 0.02) and *C002* (*F*_(2,6)_ = 6.16, *P* = 0.04) in salivary glands decreased significantly after 72 h of feeding (Fig. 2A and B). *ACYPI006346* gene expression showed a similar trend but was not statistically significant (*F*_(2,6)_ = 3.72, *P* = 0.09) (Fig. 2C).

**Fig. 3.**
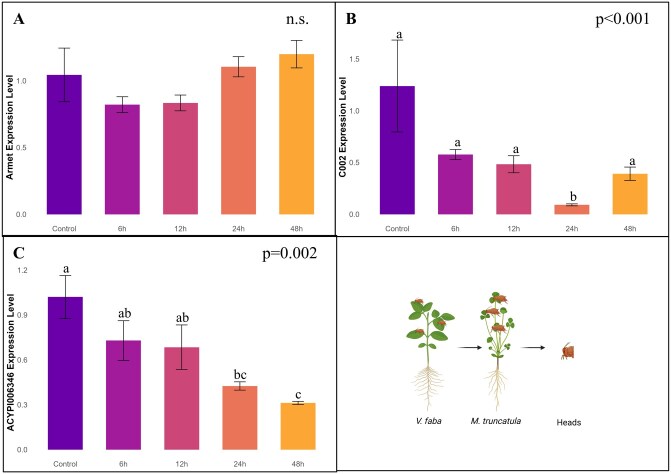
Short- to intermediate-term gene expression dynamics in aphid heads after host switch. Relative gene expression profiles of A) *Armet*, B) *C002*, and C) *ACYPI006346* in pea aphid heads (*Acyrthosiphon pisum*) at 0 (taken directly from *V. faba*), 6, 12, 24, and 48 h following transfer from *Vicia faba* to *Medicago truncatula*. Relative gene expression was normalized to the reference gene *SDHB*. Data represent mean ± SEM (3 biological replicates). Each biological replicate consisted of 20 dissected aphid heads. Letters denote significant differences across time points (Tukey’s HSD, *P* < 0.05).

To capture early-stage gene expression changes post host switch, experiment B consisted of shorter time points (0 to 48 h) and included dissected aphid heads. Gene expression of *C002* (*F*_(4,10)_ = 11.42, *P* < 0.001) was downregulated exclusively at 24 h, whereas gene expression of *ACYPI006346* (*F*_(4,10)_ = 9.02, *P* = 0.002) was downregulated both at 24 and 48 h (Fig. 3B and C). *Armet* gene expression (*F*_(4,10)_ = 2.03, *P* = 0.17), however, remained stable across the time course (Fig. 3A).

### Gene Expression of Pea Aphid Salivary Effectors Slightly Fluctuates When Aphids Feed on a Single Host

**Fig. 4.**
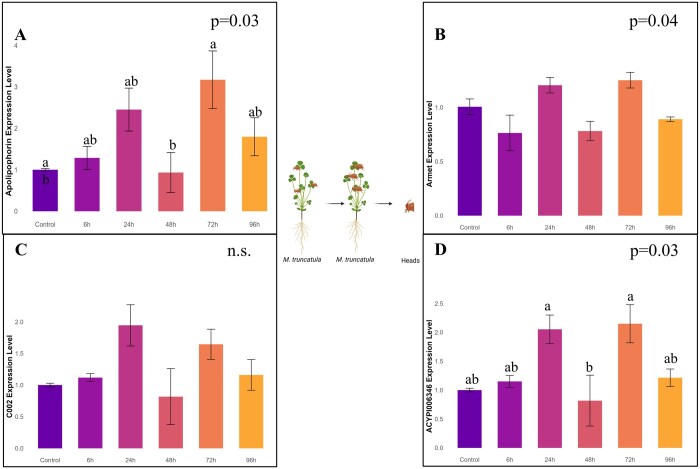
Time course of salivary effector gene expression in aphid heads on a single host. Relative gene expression of A) *Apolipophorin*, B) *Armet*, C) *C002*, and D) *ACYPI006346* in pea aphids (*Acyrthosiphon pisum*) reared and continuously fed on *Medicago truncatula*. Samples were collected at 0 (taken directly from *M. truncatula*), 6, 24, 48, 72, and 96 h. Relative expression was normalized to the geometric mean of the reference genes *Rps20* and *TpL27*. Data represent mean ± SEM (3 biological replicates). Each biological replicate included 5 dissected aphid heads. Letters denote significant differences across time points (Tukey’s HSD, *P* < 0.05).

In experiment C, aphids were reared and fed continuously on *M. truncatula* and were sampled from 0 (taken from *M. ­truncatula*) to 96 h to assess gene expression patterns. Gene expression of *Armet* (*F*_(5,12)_ = 3.48, *P* = 0.04), *Apolipophorin* (*F*_(5,12)_ = 3.61, *P* = 0.03), and *ACYPI006346* (*F*_(5,12)_ = 3.90, *P* = 0.02) showed significant fluctuations between specific time points (Fig. 4A, B, and D). *Apolipophorin* and *ACYPI006346* gene expression was upregulated at 72 h compared to the 48 h time point (Fig. 4A and D). Additionally, *ACYPI006346* gene expression was upregulated at 24 h compared to the 48 h time point. Overall, all time points showed similar gene expression patterns than the 0 h control (Fig. 4D). *Armet* gene expression was statistically significant (*F*_(5,12)_ = 3.48, *P* = 0.04); however, Tukey’s HSD test did not identify significant pairwise differences among individual time points (Fig. 4B). Notably, *C002* gene expression remained relatively stable (*F*_(5,12)_ = 2.51, *P* = 0.09) (Fig. 4C).

### Salivary Effector Gene Expression is Reduced in 6-Day Old Aphids Feeding on a Single Host

**Fig. 5.**
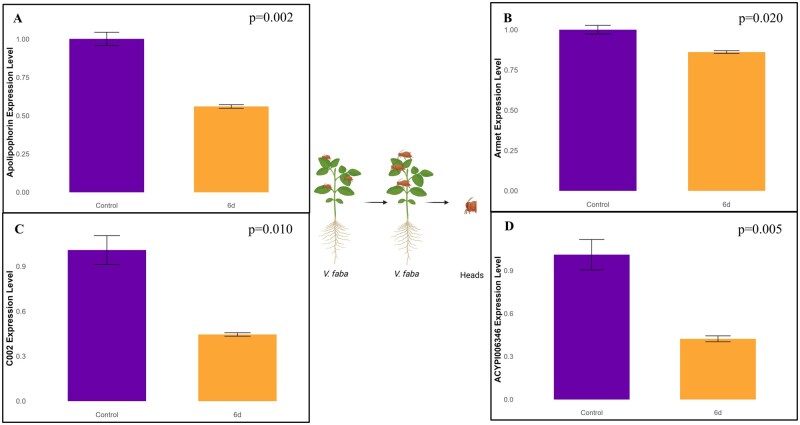
Effect of aphid age on salivary effector gene expression. Relative gene expression of A) *Apolipophorin*, B) *Armet*, C) *C002*, D) *ACYPI006346* in 1-day-old and 6-day-old pea aphids (*Acyrthosiphon pisum*) that continuously fed on *Vicia faba*. Relative expression was normalized to the geometric mean of the reference genes *Rps20* and *Actin*. Data represent mean ± SEM (3 biological replicates). Each biological replicate consisted of 20 dissected aphid heads. A *P* value less than 0.05 denotes statistically significant differences.

In experiment D, we assessed whether pea aphid developmental stage influences effector gene expression. Here, we compared 1-day-old and 6-day-old aphids feeding on *V. faba*. All 4 tested salivary effector genes, *Armet* (*t*_(2.55)_ = 5.25, *P* = 0.020), *C002* (*t*_(2.27)_ = 7.95, *P* = 0.010), *Apolipophorin* (*t*_(2.84)_ = 11.92, *P* = 0.002), and *ACYPI006346* (*t*_(2.83)_ = 7.53, *P* = 0.006), were significantly downregulated in 6-day-old aphids (Fig. 5A to D).

## Discussion

The main goal of this study was to identify the key factors, namely, host plant, aphid age, and feeding duration, that influence the expression of aphid salivary effector genes. Our findings offer foundational insights for designing more standardized and interpretable transcriptomic experiments involving aphids. While many omics studies have explored aphid salivary effectors, the lack of consensus regarding sampling strategies, including tissue type, developmental stage, plant condition, and feeding duration, has hindered cross-study comparisons (Bos et al. 2010, Thorpe et al. 2016, 2018, Zhang et al. 2017, Boulain et al. 2018, 2019, Wang et al. 2020). Our approach addresses this gap by systematically evaluating experimental variables that are often overlooked in transcriptome studies.

Our results revealed that aphid salivary effector gene expression is dynamic, varying with feeding time and age. The first hypothesis states that shifting pea aphids from *V. faba* to *M. truncatula* is expected to trigger a rapid, transient upregulation of key salivary effector genes (*Armet*, *C002*, *ACYP1006346*) during early host acceptance, followed by a partial decline toward a new steady state as aphids establish sustained feeding on the new host. Among the 4 salivary effectors tested, *C002*, *Armet*, and *ACYPI006346* exhibited the most dynamic gene expression profiles at 24 h (*C002* and *ACYPI006346*), 48 h (*ACYPI006346*), and 72 h (*C002* and *Armet)*, especially when switching hosts, supporting the first hypothesis (Figs 2A to C and 3A to C). However, we did not observe upregulation of salivary effector genes when switching hosts. Notably, the use of aphid head samples offered a practical compromise for time course experiments, allowing successful detection of the salivary gland marker *ACYPI006346* (Figs 3C, 4D, and 5D). Interestingly, *ACYPI006346* transcript levels vary in 3 *A. pisum* colonies adapted to *V. faba*, *M. truncatula*, and *V. villosa*, but its function in aphid–plant interactions is still unclear (Pan et al. 2015). Our findings align with previous studies showing that salivary effector expression is temporally regulated and may be repressed once aphids establish feeding sites (Xue et al. 2024). Electrical penetration graph analyses support this timing, with aphids exhibiting more stable feeding behaviors after approximately 5 h on new hosts (Lu et al. 2016). Interestingly, *Armet* has also been linked to elevated salicylic acid signaling in plants without reducing aphid fitness (Cui et al. 2019; Canham 2022), suggesting it may modulate the host environment to benefit aphid feeding. Despite triggering defenses, aphids showed a preference for *Armet*-infiltrated leaves, highlighting the nuanced role of effectors in manipulating host physiology (Lu et al. 2016).

*C002* was first identified as the most abundant cDNA from an *A. pisum* salivary gland cDNA library and is commonly used as a positive control in RNA interference studies (Mutti et al. 2006, 2008, Pitino et al. 2011). Interestingly, we found that *C002* gene expression decreases at specific feeding times (Figs 2B, 3B, and 5C). A recent study that examined the diurnal *C002* gene expression in bird cherry-oat aphids (*Rhopalosiphum padi*) found that its expression peaks at midnight, which may help explain the results of the present study (Han et al. 2024). In addition, gene expression of salivary effectors varies significantly among different aphid biotypes, suggesting that gene expression is influenced more by the aphid line and biotype than by the host plants (Boulain et al. 2019). These gene expression patterns reinforce the idea that effector deployment is tightly linked to feeding stage and plant compatibility.

The second hypothesis stated that prolonged feeding on *M. truncatula* will drive dynamic changes in aphid salivary effector gene expression, reflecting progressive physiological adaptation to the new host. Our data showed upregulation of *Apolipophorin* and *ACYPI006346* at 72 h compared to the 48 h time point (Fig. 4A and D). However, all time points showed similar gene expression patterns to the 0 h control (Fig. 4D), therefore not fully supporting the hypothesis. Han et al. (2024) observed diurnal rhythmicity in the expression patterns of salivary effectors in the bird cherry-oat aphid (*R. padi*). Exploring diurnal gene expression patterns in pea aphids could clarify whether temporal dynamics occur.

The third hypothesis stated that younger aphids will exhibit higher salivary effector gene expression than older aphids, reflecting distinct metabolic and developmental demands early in life. Indeed, we found that gene expression of the 4 salivary effectors tested was higher in 1-day-old aphids (Fig. 5A to D). Younger aphids likely require enhanced effector activity to facilitate early feeding and colonization. Studies in other aphid species, such as Indian grain aphid (*Sitobion miscanthi*, Takahashi) corroborate this idea, showing peak effector expression during initial feeding stages (Fu et al. 2023).

Together, these findings highlight the need for methodological consistency in transcriptomic studies. Future work should emphasize well-replicated, time-resolved designs that account for tissue type, developmental stage, and host condition to improve reproducibility and enable robust meta-analyses. A key limitation of our temporal feeding assays (experiments A to C) is the difficulty of separating aging effects from feeding duration, as aphids age throughout the time course. For example, aphids in the 96 h time point were 4 days older than in the 0 h controls (experiment C). Although experiment D showed that age independently influences gene expression, future studies should use age-staggered cohorts introduced to hosts at different times so all individuals can be harvested at the same chronological age. This would isolate transcriptional responses to host adaptation from developmental progression. Investigating the mechanisms driving rhythmic effector expression—and whether these rhythms can be disrupted to impair feeding—also represents a promising direction. Ultimately, clarifying how aphids adjust their secretome in response to plant cues and internal physiology may inform new pest-management strategies.

## Conclusions

This study highlights the plasticity of salivary effector gene expression in pea aphids and the importance of experimental design in transcriptomic research. We demonstrate that feeding duration and aphid age independently impact salivary effector gene expression patterns. These findings lay the groundwork for future functional analyses and provide a roadmap for more standardized, replicable studies of aphid–plant interactions. By refining sampling strategies and aligning protocols across studies, researchers can more accurately capture the complex molecular dialogues between aphids and their host plants.

## Supplementary Material

ieag056_Supplementary_Data
