# Supplementary material for: Gene expression of pea aphid (Hemiptera: Aphididae) salivary effectors changes based on feeding duration and plant species
Source: J Insect Sci. 2026 Jun 19;26(3):ieag056. doi: 10.1093/jisesa/ieag056 (PMC13283480; doi:10.1093/jisesa/ieag056)
Supplement: ieag056_Supplementary_Data [file ieag056_supplementary_data.docx]

**Supplementary Table S1. Oligonucleotide details for reference and salivary effector genes and associated RT‑qPCR conditions.**

| **NCBI** | **Gene** | **Oligonucleotide sequence (5′–3′)** | **Annealing Temp (°C)** | **Efficiency (%)** | **Stability Value** | **Reference** |
| --- | --- | --- | --- | --- | --- | --- |
| **Reference genes** | | |  |  |  |  |
| NM_001162436 | *SDHB* | F: CTGAATTCCTGTGGACCTATGG  R: ACGGCAAGAACGCCTAAA | 62.0 | 99.1 | 0.120 | Yang et al., 2014 |
| NM_001162819.2 | *Rps20* | F: AAGTGTGTGCTCCGAGATGA  R: CAGCAATGACACCGGGTTC | 63.2 | 98.8 | 0.153 | Niu et al., 2019 |
| NM_001126200 | *Actin* | F: CGTTACCAACTGGGACGATATG  R: GGGTTCAATGGAGCTTCTGTTA | 62.5 | 90.3 | 0.189 | Yang et al., 2014 |
| CN584974 | *TpL27* | F: CCGAAAAGCTGTCATAATGAAGACC  R: GGTGAAACCTTGTCTACTGTTACATCTTG | 64.6 | 96.9 | 0.225 | Mutti et al., 2006 |
| **Salivary effector genes** | | |  |  |  |  |
| LOC100167188 | *Armet* | F: GTTACTATTTGGGCGGATTA  R: TATCGCAGACTTGAGCAT | 57.9 | 95.9 | NA | Pan et al. 2015; Cui et al. 2019 |
| LOC1001167863 | *C002* | F: CTCACTCGCCTACCCTTTCG  R: CGTAGTGAGTGGTGAGCCAG | 63.8 | 93.5 | NA | Mutti et al. 2008 |
| LOC100259010 | *Apolipophorin* | F: CTGACCCATCGTTTGCTGGA  R: TCAACCGTAAAAGTAGAACCATCG | 63.6 | 96.9 | NA | Zdybicka-Barabas et al. 2013 |
| *AK341542* | *ACYPI006346* | F: ATCCAGTCGATTGTCCGTCC  R: ACGCTTGGCGGTATTTAAGC | 63.4 | 97.8 | NA | Pan et al. 2015 |

**Note:** In experiments A and B, *SDHB* was used as the reference gene, while in experiment C, Tp*L27* and *Tps20* were used, and in experiment D, *Actin* and *Rps20* served as the reference genes. The NormFinder software (https://www.moma.dk/software/normfinder) was used to determine the stability values for reference genes. All salivary effector gene primers were designed using NCBI primer-BLAST.
